# Chlamydia Species and Related Risk Factors in Poultry in North-Western Italy: Possible Bird-to-Human Transmission for *C. gallinacea*

**DOI:** 10.3390/ijerph19042174

**Published:** 2022-02-15

**Authors:** Monica Marchino, Francesca Rizzo, Paola Barzanti, Oriana Anna Sparasci, Paolo Bottino, Nadia Vicari, Sara Rigamonti, Silvia Braghin, Rachid Aaziz, Fabien Vorimore, Giuseppe Ru, Karine Laroucau, Maria Lucia Mandola

**Affiliations:** 1 Experimental Zooprophylactic Institute of Piedmont, Liguria and Aosta Valley (IZSPLV), Via Bologna 148, 10154 Turin, Italy; fragiol@libero.it (F.R.); paola.barzanti@izsto.it (P.B.); oriana.sparasci@izsto.it (O.A.S.); giuseppe.ru@izsto.it (G.R.); 2 S.C. Microbiology and Virology Unit, Azienda Ospedaliero Universitaria “Città della Salute e della Scienza di Torino”, 10126 Turin, Italy; paolo.bottino@unito.it; 3 National Reference Laboratory for Chlamydioses, Experimental Zooprophylactic Institute of Lombardia and Emilia Romagna (IZSLER), 27100 Pavia, Italy; nadia.vicari@izsler.it (N.V.); sara.rigamonti@izsler.it (S.R.); 4 S.C. Sanità Animale, Dipartimento Di Prevenzione, ASL CN1, Via Carlo Boggio 12, 12100 Cuneo, Italy; braghin.silvia@aslto5.piemonte.it; 5 Laboratory for Animal Health, Bacterial Zoonosis Unit, ANSES Maisons-Alfort, Paris-Est University, 94706 Paris, France; rachid.aaziz@anses.fr (R.A.); fabien.vorimore@anses.fr (F.V.); karine.laroucau@anses.fr (K.L.)

**Keywords:** *Chlamydiaceae*, *Chlamydia gallinacea*, Italy, poultry, risk factors, *One Health*, *Chlamydia psittaci*, zoonosis, bird-human transmission

## Abstract

*Chlamydiaceae* are obligatory intracellular bacteria causing acute and chronic diseases in animals and humans worldwide, with recently discovered species with a still unclear pathogenic potential (i.e., *C. gallinacea*). In Italy, *Chlamydiaceae* infections are underestimated both in animals and humans. To estimate the prevalence of *Chlamydiaceae* species in poultry and occupationally exposed workers on farm, a cross-sectional study was carried out in north-western Italy. A total of 2063 samples from 83 commercial and 31 backyard poultry farms were analysed using real-time PCRs for *Chlamydiaceae* screening and species typing. *Chlamydiaceae* were detected in 23 farms, with a herd prevalence of 20.2% (95%CI: 13.2–28.7), higher in backyard farms (38.7%; 95%CI: 21.8–57.8) compared to commercial ones (13.3%; 95%CI: 6.8–22.5). *C. gallinacea* was found in 18 chicken farms, both commercial and backyard, and *C. psittaci* only in 3 backyard farms. Exposure to wild birds and factors related to biosecurity resulted the main risk factors associated with *Chlamydia* positivity. Out of the 113 sputum samples collected from farmers, 16 tested positive to *Chlamydiaceae,* with a prevalence of 14.2% (95%CI: 8, 3–22). To the best of our knowledge, for the first time at international level, *C. gallinacea* was detected in humans with farmer positivity associated with farm infectious status, suggesting a bird-to-human transmission.

## 1. Introduction

The obligate intracellular bacteria *Chlamydia* (spp.) are the aetiological agents of chlamydiosis in wild and domestic birds, mammals, and humans [1]. Due to their intrinsic high genetic diversity, the taxonomic classification within the family *Chlamydiaceae* in the *Chlamydiales* order is constantly evolving following the identification of new chlamydial strains, mainly of avian origin. According to very recent findings in flamingos, the family *Chlamydiaceae,* so far composed by the single genus *Chlamydia,* including 14 recognised species, appears to be enriched with a new proposed genus *Chlamydiifrater* gen. nov., including two new species named *Chlamydiifrater phoenicopteri* sp. nov. and *Chlamydiifrater volucris* sp. nov. [2,3]. As for the genus *Chlamydia*, in addition to the old ones (i.e., *C. trachomatis*, *C. suis, C. muridarum*, *C. pneumoniae*, *C. abortus*, *C. caviae*, *C. felis*, *C. pecorum,* and *C. psittaci*), five new species, characterised through whole-genome sequencing (WGS), have been introduced in the last decade. Specifically, *C. avium, C. gallinacean,* and *C. buteonis* were identified in birds while *C. serpentis* and *C. poikilothermis* in snakes [4,5,6]. Additionally, new *candidate* species and taxa have been lately described in fishes and reptiles [2,7,8].

*C. psittaci,* long considered to be the only pathogenic species in birds and aetiological agent of avian chlamydiosis and human psittacosis, is common in poultry farms worldwide [9,10]. The disease severity in birds varies according to host species, age, and immune status as well as to the virulence of the bacterial strain [10,11]. In most cases, *C. psittaci* outbreaks in poultry may be characterized by mild respiratory symptoms and latent infections with intermittent and recurrent shedding of the pathogen, often leading to chronic clinical forms [11,12]. Since high load of *C. psittaci* is shed via faeces and nasal discharges, aerosol dissemination and sometimes ingestion of contaminated material are the main routes of chlamydial transmission [13]. Other ways of transmission include sharing of contaminated water sources and bloodsucking ectoparasites’ bites; moreover, vertical transmission has been proven in poultry and some wild bird species [14,15]. Thus, birds act as carriers and important reservoirs of infection, posing a potential threat to both other animal species and humans [11,16,17,18].

Psittacosis, defined as the human disease caused by *C. psittaci* zoonotic infection, is an occupational disease, affecting mostly bird handlers, veterinarians, poultry workers, and slaughterhouse workers who are exposed to the highest risk of infection by manipulating or having contact with infected birds and fomites [10]. Symptoms in humans range from asymptomatic infection to severe and/or systemic disease affecting multiple organ systems with fever, headache, respiratory disease, and other manifestations (including endocarditis, myocarditis, hepatitis, arthritis, conjunctivitis, encephalitis) [11,19,20].

Nevertheless, a much more complex epidemiology for avian chlamydiosis has been disclosed, suggesting that species other than *C. psittaci* may also be involved in the aetiology of the disease in birds, including *C. gallinacea*, *C. avium*, *C. abortus*, C. *pecorum,* and *C. trachomatis* [4,21,22]. *C. gallinacea* appears to be widely distributed worldwide and has been reported as the predominant agent of chlamydiosis in poultry in Argentina, China, the Netherlands, Poland, the USA, Australia, and Mexico [4,23,24,25,26,27,28,29]. Since 2008, this new species has also been randomly detected in asymptomatic poultry in northern Italy (including Piedmont and Liguria regions) [30,31]. Moreover, a study conducted in 2018 on 160 rural free-range chicken farms recorded a PCR prevalence of 15% for *C. gallinacea* in several regions of Italy, with the isolation of eight strains [32]. By molecular typing on at least 25 strains, *C. gallinacea* appears as a high diverse species accounting for at least 13 *omp*A types and 15 sequence types (ST) [23,33].

While the zoonotic potential of *C. gallinacea* was first evoked after its identification following atypical cases of pneumonia in a French slaughterhouse [34], no case has been confirmed yet. More recently, a serological study conducted in Poland showed that almost 20% of exposed individuals, all farmers or farm workers, were seropositive for *Chlamydiaceae.* Unfortunately, due to the lack of species-specific serological methods, the study did not allow the identification of the humoral immune response to *C. gallinacea* specifically [26]. In the Netherlands, an investigation set up on throat swabs from farmers working in *C. gallinacea*-positive poultry farms could not detect any human infections by real-time PCR [27]. Therefore, pathogenicity and possible zoonotic potential of *C. gallinacea* have yet to be systematically investigated.

Chlamydial infections continue to be underestimated and underreported in both poultry and human sectors worldwide [35,36]. To date, the infection is not routinely investigated as part of the diagnosis panel in case of respiratory diseases and pneumonia in humans [36]. In Italy, avian chlamydiosis due to *C. psittaci* infection is included in the animal notifiable diseases list, and psittacosis is included among notifiable occupational diseases.

The Italian poultry sector is a completely self-sufficient system, producing more than what Italy consumes, with a self-supply rate of about 108% [37]. Poultry farming is practiced throughout the country, but it is particularly concentrated in northern regions. The Italian poultry farming scenario is made up of 15,300 farms in production, of which over 6000 are professional, employing almost 40,000 workers, and 1600 slaughtering, cutting, or egg processing plants, accounting for 25,500 employees. In Europe, Italy is the sixth largest poultry meat producer and the third largest eggs producer. Almost all of the Italian poultry production consists of an integrated chain, of which almost 90% is managed by big companies [38].

To date, studies on chlamydial agents are strategic due to their potential health and economic impact on poultry and humans. The project was undertaken with the aim of (i) investigating prevalence and diversity of *Chlamydia* spp. through a cross-sectional study, (ii) exploring potential risk factors in commercial and backyard poultry farms, and (iii) exploring potential risk factors in professionally exposed workers in the study area. Moreover, the study aimed at promoting more effective monitoring and reporting activities applying a *One Health* approach, as recommended at international level.

## 2. Materials and Methods

### 2.1. Study Design and Sampling Strategy

**Figure 1:**
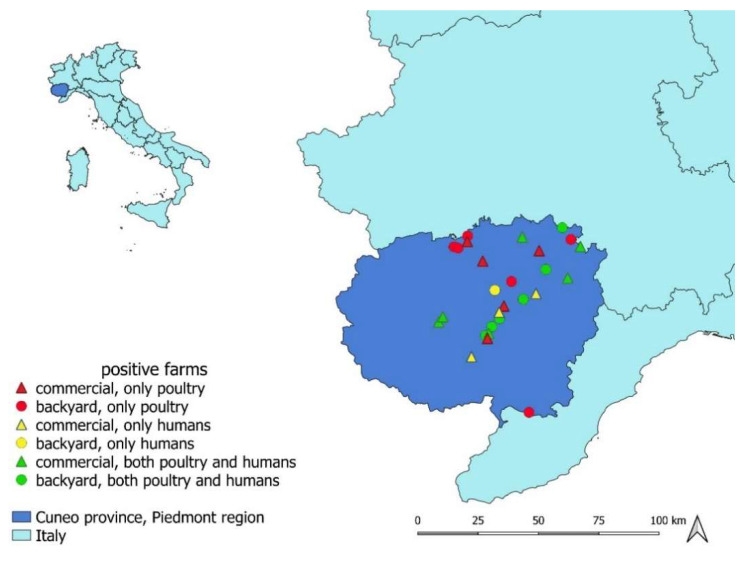
Map of the *Chlamydiaceae*-positive farms plotted on the map of Cuneo province (Piedmont region, north-western Italy), where the current cross-sectional study was conducted. Farms in which only poultry, only humans, or both poultry and humans tested positive to *Chlamydiaceae* PCR screening are indicated with different colours and symbols.

From May 2018 to January 2021, a cross-sectional study was performed on poultry farms in Cuneo province (Figure 1). This is the area with the highest poultry farm density in Piedmont region, accounting for approximately 420 commercial and 100 backyard farms, according to the National Data Bank (BDN) of livestock register (National Data Bank of the Veterinary Information System, Ministry of Health, March 2018) [39]. Due to the sharing of some risk factors, whenever possible, samples collection was carried out as part of the Avian Flu and Salmonella surveillance programmes in poultry farms.

In the target population of domestic poultry as present in BDN, the study involved a two-stage sampling plan. First of all, farms (primary sample units, PSU) were appropriately stratified according to: (i) holding size (large-scale commercial with >250 animals raised or backyard farms with <250 animals raised) and, only for large-scale commercial, (ii) species reared (chicken, duck, turkey, geese, or mixed poultry) and (iii) type of farming (weaners, layers, broilers, breeders). At the first stage, to calculate the PSU sample size aimed at estimating inter-herd *Chlamydia* prevalence, an expected prevalence (P) of 0.26 [40], an error of 0.10, a confidence level of 0.95, and the single-layer population size were considered. In each layer, farms were randomly selected; where this was not possible because of the smallness of the layer, all the farms present in it were considered. At the second stage, within each PSU a simple random sampling of animals (minimum sample size identified: *n* = 16 for commercial farms; *n* = 15 for backyard farms) was carried out, aimed at detecting a within-herd prevalence of 0.20 (design prevalence), assuming an animal-level sensitivity of 0.95, a specificity of 1.00, and a confidence level of 0.95. All the appropriate sample sizes were identified by means of Epitools (Copyright © 2022 AusVet) [41].

To define the role of potential risk factors associated with the presence of *Chlamydia* in poultry farms, data on structural characteristics, management, and farm location were collected through an epidemiological questionnaire, containing 33 closed-ended questions and filled in by a collecting veterinarian.

With the aim of studying potential zoonotic transmission of new *Chlamydia* species associated with occupational exposure to poultry, farmers and farm workers as well as veterinary officers were asked to voluntarily take part in the study by submitting and self-sampling their sputum. A self-administered questionnaire to collect individual anamnesis of the enrolled subjects was set up. Questions were related to biographical, anamnestic, and clinical information at the time of sampling as well as to the work activity. A cover letter presenting the objectives of the study was attached to the questionnaire together with a form to allow the processing of personal data in accordance with privacy regulations and instructions for sputum self-collection.

The study involved the collection of different types of samples: two dry cloacal swabs were taken from each live animal, placed in the same sterile tube, and kept at a 4 °C until the delivery to the laboratory. Sputum was collected from humans in a sterile container and kept at a −80 °C until analyses.

### 2.2. Laboratory Analyses

#### 2.2.1. Nucleic Acid Extraction

DNA isolation from samples was performed as follows:

For cloacal swabs: upon arrival at the laboratory (within 48 h after collection), 2 mL of SPG buffer (liquid medium capable of preserving *Chlamydiaceae* viability) were added to the tube [9]. After centrifugation (1000× *g*/10′), total nucleic acids in the supernatant were purified using Maxwell^®^ RCS Viral Total Nucleic Acid Purification Kit (AS1330), in Maxwell^®^ RCS 48 instrument, according to the manufacturer’s instructions. Elution volume was set at 100 µL.

For human sputum: upon arrival at the microbiology laboratory, samples were immediately stored at −80 °C for subsequent analysis. After thawing, each sputum sample was dissolved with Sputasol buffer (ThermoFisher Scientific, Waltham, MA, USA) at a 1:1 ratio. Subsequently, extraction of bacterial DNA was performed on platform QIAsymphony SP/AS (Qiagen, Hilden, DE) using the DSP Virus/Pathogen Kit (Qiagen, Hilden, DE). The input volume was related to the starting volume of the sample (from 500 µL to 1 mL), while the elution volume was 110 µL.

#### 2.2.2. Screening and Typing of Chlamydia Species

DNA samples were firstly screened for the ribosomal 23S gene (highly conserved within the family *Chlamydiaceae*) by a *Chlamydiaceae*-specific real-time PCR [42]. All positive DNA samples were further analysed with species-specific real-time PCR assays (i.e., species-typing) targeting the *omp*A gene for *C. psittaci* and *C. abortus* [21] and the *eno*A gene for *C. avium* [43] and *C. gallinacea* [17].

The amplification of the genomic material was performed using GoTaq^®^ Probe qPCR Master Mix by Promega (Madison, WI, USA); for animal samples, the thermal cycle used on CFX96 Instrument (BIORAD, Hercules, CA, USA) for the species-typing was the following: 95 °C for 2 min, 95 °C for 15 s, and 60 °C for 1 min × 45 cycles, while for human samples, qPCR reactions were performed on the ABI7500 Instrument (Applied Biosystems, Waltham, MA, USA) using the above-mentioned thermal profile. Samples with a cycle threshold (Ct) values ≥ 40 were considered negative for each real-time PCR assays.

#### 2.2.3. Data and Statistical Analyses

Veterinary and human data obtained as a result of laboratory investigations as well as information collected through the epidemiological questionnaires were entered in two ad hoc databases (provided as supplementary material 1) in which anamnestic, epidemiological, and laboratory data relating to each enrolled farm and to the samples collected (animal/human) were recorded. In the first database (veterinary data), the elementary epidemiological unit was the farm; in the second database (human data: farmers and farm workers, veterinary officers), it was the single sampled individual. Since they took part in the research on a voluntary basis, in 24 of the selected farms, no human samples were collected; on the other hand, in the other farms, from 1 to 8 individuals were sampled; therefore, in the second dataset, some farm codes are repeated. Animal and human data were matched, whenever possible (veterinary officers’ data, given that they work on the territory and cannot be linked to a single specific farm code, were analysed separately and only in relation to individual risk factors), using the unique key defined by the farm code.

A farm was considered positive when at least one animal tested positive. The data collected allowed to calculate point and interval (exact binomial) prevalence estimates. Inter-herd prevalence estimates both crude and by holding size category were produced and compared using the chi-square test, while within-herd prevalence estimated as the median value of the percentage of tested positive animals (and the values of the first and third quartile Q1–Q3) was calculated only for descriptive purposes. Contingency tables (bivariate analyses) have been used for the calculation of relative risks, in terms of prevalence ratios (PRs) of exposed to non-exposed, to identify putative risk factors potentially associated with the positivity of the farm. A PR value was considered statistically significant if its 95%CI did not overlap 1. Risk factors statistically associated to the risk or close to the statistical significance in the bivariate analysis were included in a multivariate Poisson regression model, using the farm as the epidemiological unit.

For human data, bivariate PRs were calculated to identify candidate factors associated with the risk of positivity in occupational-exposed workers: the host- and farm-level putative risk factors were included in a preliminary mixed-effects Poisson regression model to account for the potential random effect associated to farm. After confirming its absence, a final multivariate Poisson regression model was used.

The Stata SE (version 16.1) software was used for statistical processing of both veterinary and human data (College Station, TX: StataCorp LLC).

#### 2.2.4. MLST Typing

Genotyping by MLST was carried out according to the scheme developed by Pannekoek and colleagues [44], targeting seven housekeeping genes: *gat*A, *opp*A, *hfl*X, *gid*A, *eno*A, *hem*N, and *fum*C. Target genes were amplified and sequenced using primers and conditions described for *C. gallinacea* by Guo and colleagues [33]. A dendrogram was constructed with the software MEGA7 using the Neighbor-Joining method. New allele sequences are accessible via the Chlamydiales MLST web-site [45] (http://pubmlst.org/chlamydiales/ (accessed on 16 September 2021)).

## 3. Results

In the study period, 114 farms were visited: 31 backyard farms and 83 large-scale commercial farms. Sample collection for *Chlamydiaceae* presence investigation involved a total of 2063 domestic birds and 145 occupational-exposed workers, including farmers, farm workers, and veterinary officers.

### 3.1. Poultry Samples

A total of 2063 cloacal samples were collected from domestic poultry, 1518 of which were from 83 commercial farms and 545 from 31 backyard farms, representing 73 and 27% of the total number of sampled farms, respectively. The farms were characterized by reared species and type of farming, as detailed in Table 1.

**Figure 2:**
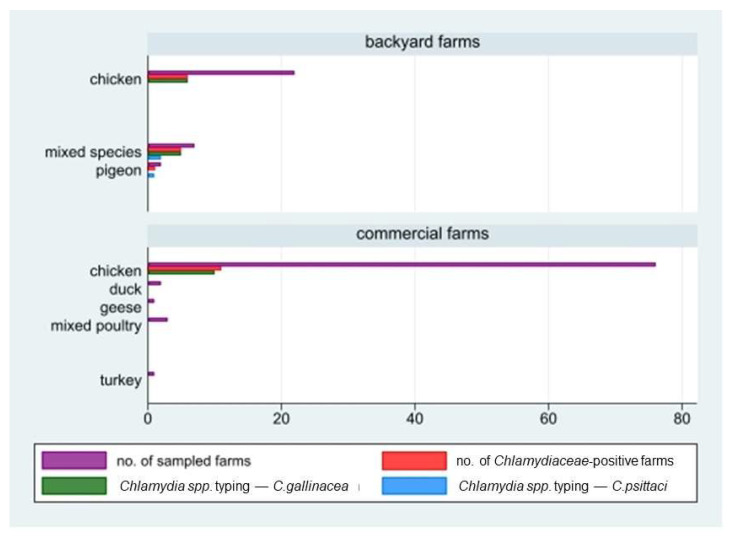
Number of farms in study by holding size (backyard/commercial farms), reared species, and laboratory results (positivity to *Chlamydiaceae* screening and species typing; *C. gallinacea* and *C. psittaci*).

**Figure 3:**
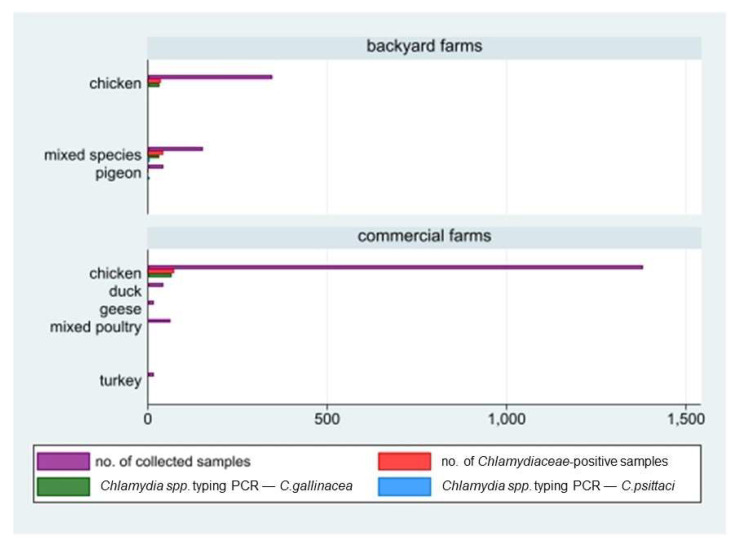
Number of animal samples collected by holding size (backyard/commercial farms), reared species, and laboratory results (positivity to *Chlamydiaceae* screening and species typing: *C. gallinacean* and *C. psittaci*).

Out of the 114 sampled farms, *Chlamydiaceae* DNA was detected in 23 of them, corresponding to an overall herd prevalence of 20.2% (95%CI: 13.2–28.7). In detail, the prevalence in large commercial farms with 11 positive farms out of 83 (13.3%; 95%CI: 6.8–22.5) was statistically lower (chi-square test, *p* = 0.0026) than that in backyard farms (12 out of 31 tested, 38.7%; 95%CI: 21.8–57.8). All commercial farms that tested positive reared chickens, while positive backyard farms reared all the species involved in the study, i.e., chickens, mixed species, or pigeons. The median within-herd prevalence value in the 23 positive farms was 24% (Q1 = 9.4; Q3 = 52). *Chlamydia* species typing performed by PCR on 155 positive samples showed the circulation both of *C. gallinacea* and *C. psittaci*. The former was detected in 132 samples collected in 21 positive farms (10 commercial and 11 backyard farms). The latter was detected in seven samples collected in three backyard farms: in two of them, both *C. gallinacea* and *C. psittaci* were detected. In detail, *C. gallinacea* was found only in poultry and mixed-species farms, while *C. psittaci* was found in two mixed-species farms (including ducks and chicken) and in one pigeon farm, with an inter-herd prevalence of 9.68% (3/31) in backyard farms (Figure 2 and Figure 3). More details are shown in Table 1.

### 3.2. Risk Factors Analysis for Poultry Farms

**Figure 4:**
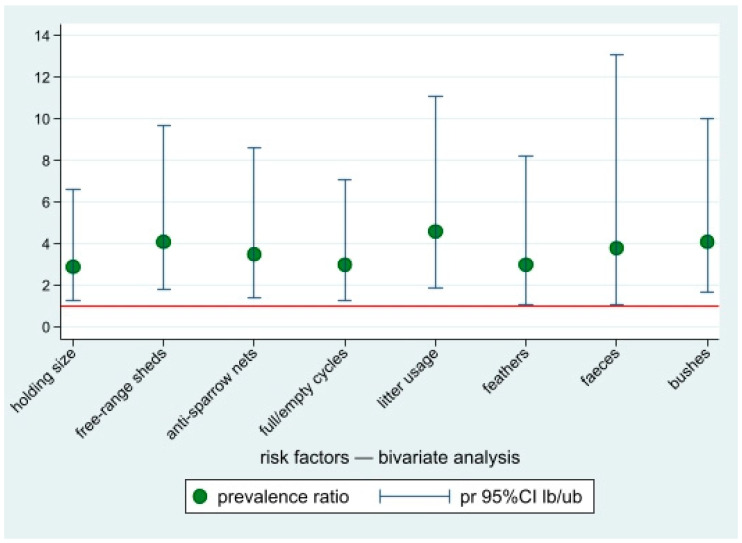
Prevalence ratios of the risk factors potentially associated with *Chlamydiaceae* positivity for occupational-exposed workers, bivariate analysis. Point estimates and lower and upper 95%CI bounds. Reference line: pr = 1.

Based on the bivariate analysis of the questionnaires data, eight factors showed an association with the risk of *Chlamydiaceae* presence in a farm, i.e., holding size (backyard vs. commercial); presence of free-range sheds (yes vs. no); presence of nets to prevent birds entry (no vs. yes); full/empty cycles (no vs. yes); litter use (no/only in free range groups vs. yes); and presence of feathers, faeces, or bushes in the surroundings of the holdings (yes vs. no) (Table 2 and Figure 4). These factors were selected as candidate covariates to fit a multivariate model.

In the multivariate Poisson regression model, only two factors were still statistically associated with the risk of farm positivity: farms where litter was only used in free-range flocks (or not regularly used) (PR = 3.6 (95%CI: 1.5–8.9)) and farms with bushes in the surroundings (PR = 3.4 (95%CI 1.4–8.5)) compared to farms with free surroundings.

### 3.3. Human Samples

**Figure 5:**
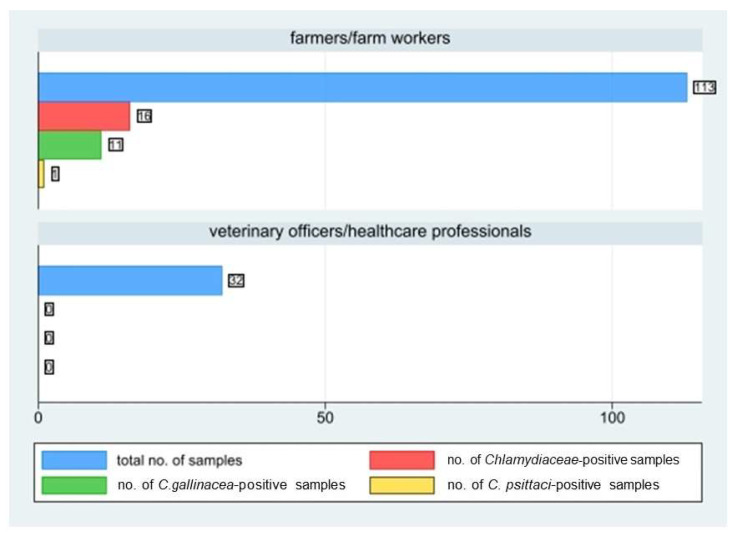
Number of human samples collected by farmers/farm workers and veterinary officers/healthcare professionals, number of positive ones to *Chlamydiaceae* screening, and to *C. gallinacea* and *C. psittaci* species-typing.

During the study, a total of 145 human samples were collected, including 113 samples from farmers or farm workers and 32 from veterinary officers and veterinary healthcare professionals. Sixteen out of the 113 farm workers (*p* = 14.2%, 95%CI: 8.3–22) tested positive to the *Chlamydiaceae* PCR screening, whereas none of the veterinary officers or healthcare professionals tested positive (Figure 5).

The prevalence in commercial farms (9 workers out of 87; 10.3%; 95%CI: 4.8–18.7) was significantly lower (chi-square test: *p* = 0.03) than in in backyard farms (7 out 26; 26.9%, 95%CI: 11.6–47.8).

In 12 farms (6 commercial and 6 backyard farms) both animals and farm workers tested positive to *Chlamydiaceae* PCR (Table 3). We did not detect more than one person positive in the same farm. *Chlamydia* species-typing revealed *C. gallinacea* in 11 out of 16 human positive samples and *C. psittaci* in only 1 human sputum. In 8 out of these 11 positive human cases, *C. gallinacea* was confirmed also at farm level. In 3 out of the 11 human cases positive for *C. gallinacea*, the farm in which the positive subject was working resulted negative for the presence of *Chlamydiaceae*.

No association between positivity and clinical signs in humans emerged in our study. For what concerns the personal protective equipment (PPE), 31% (*n* = 27) of farmers/farm workers operating in commercial farms declared not to use it compared to the 88% (*n* = 23) of those who work in backyard farms.

### 3.4. Risk Factors Analysis for Occupational-Exposed Humans

**Figure 6:**
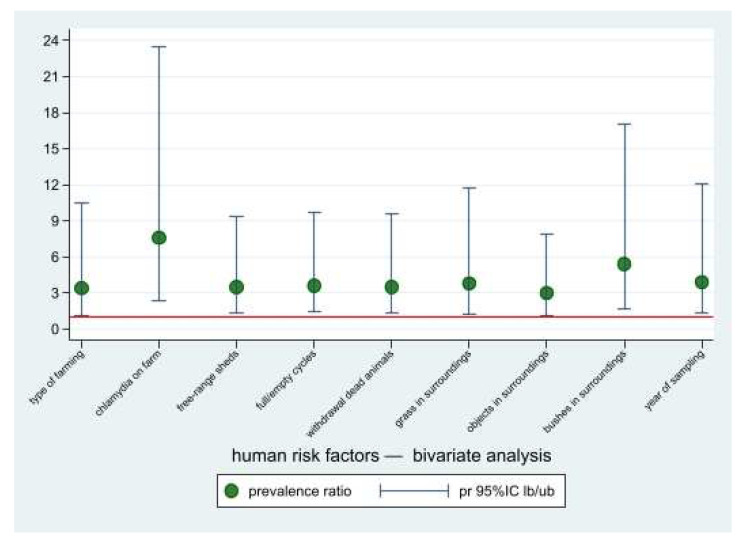
Prevalence ratios of the risk factors potentially associated with chlamydia positivity for occupational-exposed workers, bivariate analysis. Point estimates and lower and upper 95%CI bounds. Reference line: pr = 1.

Potential risk factors associated with the risk of *Chlamydiaceae* positivity for occupational-exposed workers by means of the bivariate PRs calculation (Figure 6) are shown in Table 4. These factors were subsequently submitted to the multivariate analysis. In the final Poisson regression model, only one factor was associated to the risk of human positivity, i.e., working in a positive farm (PR = 9.5; 95%CI: 2.7–33.7).

### 3.5. MLST Typing Results

**Figure 7:**
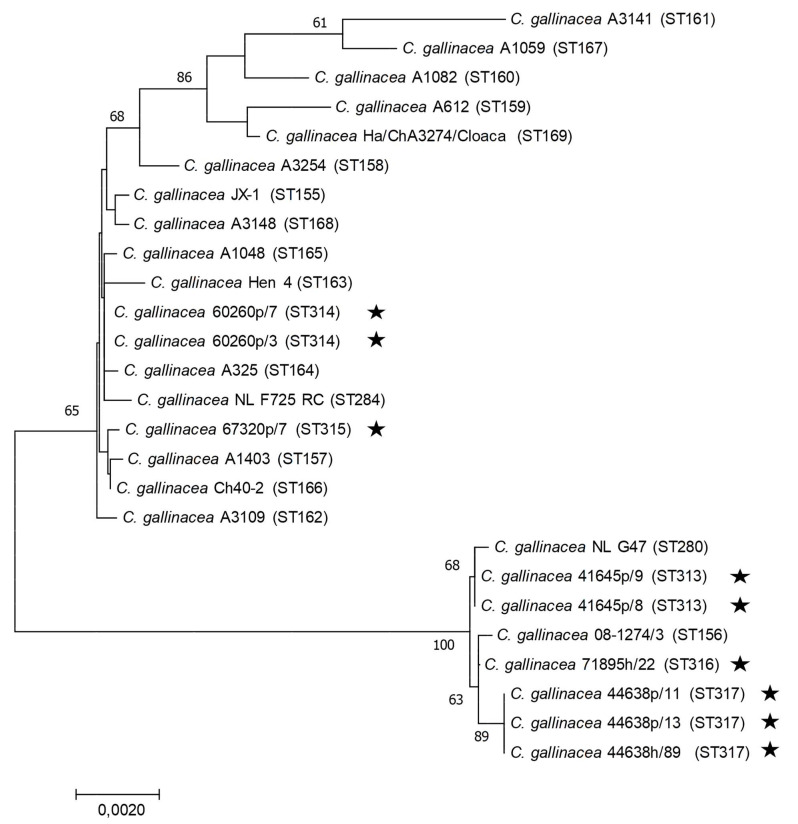
Concatenated MLST-based phylogeny of *C. gallinacea* using the Neighbor-joining method: concatenated sequences (3098 nt) were aligned and analysed in MEGA7. Phylogenetic trees were constructed using the Neighbour-Joining method and the Maximum Composite Likelihood model on all available *C. gallinacea* sequence type (ST) at [45]. Bootstrap tests were for 500 replicates. Numbers on the nodes indicate bootstrap values over 50% of the main branches. Horizontal line scale is for genetic distances. MLST sequence Type (ST) are indicated between parentheses. The star symbol represents the *C. gallinacea* samples analysed in this study.

Of the 16 human DNA samples analysed, the MLST genotyping on all seven housekeeping genes was possible only for two of them due to quality of the DNA extract. Due to the scarce quantity and quality of the DNA extracted from poultry samples, only a few DNA samples chosen within the four most affected chickens (in terms of number of positive samples and DNA concentration) were analysed by MLST typing, with a total of five MLST profiles obtained. Identical sequences were obtained each time for samples from the same farm. Except for farm 44638, for which a new sequence of the *eno*A allele was obtained, all the other gene sequences matched with sequences already present in the database, and an allele number could be assigned to each of them (Table 5). However, interestingly, the combination of alleles was new, generating new STs for these four farms. Of the 16 DNAs from human samples tested, MLST typing of 44638h_89 yielded a new ST identical to that detected on the same farm in poultry, while a new ST (again corresponding to a novel combination of alleles already described within *C. gallinacea*) was observed for 71895h_22 (no animal samples from this farm were analysed). The phylogenetic tree constructed from the concatenated MLST sequences shows a distribution of these STs among the STs identified so far from Asia and Europe, without a specific distribution that could correspond to Italy (Figure 7).

## 4. Discussion

This *One Health* study aimed to investigate prevalence and diversity of *Chlamydiaceae* in domestic poultry and in professionally exposed workers in Piedmont as well as risk factors linked to animal and human positivity.

In about one in five poultry farms in the study, *Chlamydiaceae* presence was confirmed by real-time PCR with a prevalence three times higher in backyard flocks than in commercial ones. Circulation of both *C. gallinacea* and *C. psittaci* was observed, but the former was largely more represented. *Chlamydiaceae* prevalence in poultry farms in Europe is highly variable, ranging from 6.9% in Slovakia [45] and 15.9% in Poland [26] to 47% in the Netherlands [27]. The higher prevalence in backyard than in commercial farms was expected, as previously described by Ornelas-Eusebio and coll. [29].

*Chlamydia* species-typing confirmed the circulation of *C. gallinacea* in all the backyard and commercial chicken farms resulted positive to the *Chlamydiaceae* screening but not in any mallard, turkey, geese, and mixed-poultry commercial farms. Our result is in line with recent findings describing this new chlamydial species as widespread and predominant in poultry, especially in chicken, worldwide [23,27,28,29,32].

*C. psittaci* was found only in the backyard sector, in a pigeon farm, and in two mixed-poultry farms rearing various species, including chicken and ducks. In the case of *C. psittaci*, which is able to infect more than 500 bird species from 30 different orders [16,46] and is probably ubiquitous in both domestic/companion as well as wild/free-living bird populations [47], the primary host is represented by the orders *Psittaciformes* and *Columbiformes* [12]. Nowadays, *C. psittaci* is regarded as the dominant species in pigeon and mixed-species farms and no longer represents the endemic chlamydial species in chicken (*Gallus gallus)*, being replaced by *C. gallinacea*, as described before.

In our study, two out of the three *C. psittaci* rural farms were also positive for *C. gallinacea*. Mixed infection with different chlamydial species is well known and documented [4,17,24,48,49].

*C. avium* was not detected in any poultry farm in study. In Europe, this chlamydial species has been primarily described in wild pigeons and psittacines [12,50].

In the current study, the detection of either *C. gallinacea* or *C. psittaci* was not associated with any evident sign of disease in animals [26,27,33]. This feature, already described for *C. psittaci* [10,16], has also been observed for *C. gallinacea*, suggesting a moderate pathogenicity since it did not cause any symptoms in experimentally infected chickens other than a slowdown in weight gain [23]. Consistently with that, *Chlamydia* can survive as commensal organism in the gastrointestinal tract for extended periods of time before eventually eliciting symptoms [12,51,52]. More insights on *C. gallinacea* pathogenicity have been recently reported by experimental infections in chickens, showing that infection with the NL_G47 strain does not lead to acute clinical disease after oral inoculation, and the bacteria mainly reside in the epithelium of the gut [53,54]. Nevertheless, asymptomatic infected birds may play an important role in the shedding of *Chlamydiaceae* via respiratory excretions and faeces, causing a persistent environmental contamination [10,55,56].

Almost one in ten of the human samples tested positive for *Chlamydiaceae* (one in seven if considering only farmers), with about 70% infected by *C. gallinacea.* Noteworthily, they were all poultry farm workers and farmers, while none of the veterinary officers or healthcare professionals tested positive. None of the positive farmers reported previous or ongoing signs or symptoms of respiratory illness or pneumonia. Prevalence in humans from the present study is consistent with that observed in animals from the same sampled farms. The risk was therefore associated with the status of the farm: 12 of the 16 positive farmers were working in farms where *Chlamydiaceae* had been detected. Indeed, a strong *Chlamydia*-species correspondence between animals and farmers has been highlighted although in four positive human cases, *Chlamydiaceae* presence was not confirmed in the farm where they declared to work. It should be considered that operating on more than one farm is common for farm workers, and this makes them a potential bridge for *Chlamydiaceae* spread among farms. These findings highlighted a clear link between occupational exposure and infection in humans and strongly suggest the zoonotical potential of *C. gallinacea*, hitherto only assumed [25,34,49,57]. To the best of our knowledge, this is the first description of *C. gallinacea* in occupationally exposed human specimens. In contrast to a similar Dutch study [27], which failed to detect *C. gallinacea* in human samples, our study included sputum samples instead of throat swabs. Sputum represents the most widely accepted specimen for the diagnosis of bacterial respiratory infections, and it is most likely to be (as demonstrated for *C. pneumoniae*) a better source of bacterial DNA than throat epithelium due to a higher concentration of bacteria in the deep-sited pneumonic infiltrates [58]. However, it should also be noted that in the past, primarily prior to the widespread use of molecular assays, diagnosis of psittacosis included only the targeted testing for *C. psittaci*, effectively limiting data on other *Chlamydia* species [32]. Although no association between *Chlamydia* positivity and clinical signs in humans was found in our study, the detection of *C. gallinacea* DNA in sputum from poultry workers requires attention. It must be considered that people working with *Chlamydia*-excreting poultry are likely to breathe in infectious particles, highlighting the importance of using PPE during routine activities in the farm.

Intra-species genetic diversity and phylogenetic relationships have been also investigated on a limited number of poultry and human samples. The MLST analysis confirms the genetic diversity of *C. gallinacea* by describing five new STs from different farms. Interestingly, identical STs are circulating within the same farm and not only in poultry, as demonstrated by the case of human/chicken ST identity found in the same farm. A high genetic diversity has been already described in *C. gallinacea* strains from China [33] and the Netherlands [53], but whether this feature might lead to differences in the pathogenic potential between strains is still to be determined.

While no human cases related to *C. gallinacea* have been reported in the literature since the first description of this species more than a decade ago [34], and considering the limited impact of *C. gallinacea* on poultry production [23], our results call for caution, as the consequences of a *C. gallinacea* infection in particularly vulnerable individuals (children, elderly), the immunocompromised, or clinically critical patients remain unknown.

As mentioned before, *Chlamydiaceae* prevalence appeared to be higher in backyard farms than in commercial ones, probably due to the less confined rearing conditions in backyard systems leading to an increased exposure of domestic poultry to wild birds and to the external environment that can act as source of *Chlamydiaceae* infection. In Italy, biosecurity and preventive measures have been implemented in recent years, especially in commercial farms, to counteract the entry and spread of pathogens, i.e., avian flu. Strict biosecurity measures, such as cleaning and disinfection of equipment and barn bedding, as well as feed management and preventive medicine principles represent protective factors towards pathogens introduction into the farms [29,32,55,59,60,61]. Backyard farmers are likely less aware of how to contain the risk of entry and spread of infectious diseases in their herds, mainly for the looser biosecurity regulations imposed to backyard flocks compared to commercial ones by law [61]. Moreover, in our study, they seemed less aware of the importance of PPE use in daily routine: a high percentage of backyard farmers declared not to use them in daily activities, almost three times higher than commercial ones. Because of its features, rural breeding should indeed be considered a risk factor itself for *Chlamydiaceae* presence. Since backyard chicken industry is one of the fastest growing industries in many countries of the world, the set-up of specific biosecurity rules is imperative.

In our study, the risk factor analysis showed that farms in which litter is not regularly used have a risk of positivity almost four times higher than farms in which litter is normally used. This finding is not in line with other studies in which the use of bedding material- and litter-removing practice are instead recognized as risk factors. The role played by the litter in introducing and maintaining *Chlamydiaceae* seems related to the large movements of infected dust during litter removal. Dust can represent a vehicle of bacteria, viruses, and toxins that may adhere to poultry feathers, representing a source of *Chlamydia* infection [4,29,62]. Nevertheless, You and colleagues [15] demonstrated that *C. gallinacea* can be efficiently transmitted by faecal-oral route but not via aerosol. Based on these recent observations and in relation to our findings, we can hypothesize that *Chlamydia* may be removed from the farm premises more easily and regularly if it is settled on the litter. This practice is particularly relevant in case of full/empty cycles, when stringent cleaning and disinfection are done at the end of each cycle, provided that farm workers always wear personal protective equipment.

Our study also found that farms with bushes in the surroundings had a risk of *Chlamydia* infection 3.4 times higher than those with no bushes. This may be due to a higher exposure of poultry to contact with wild birds, which may hide or nest there. Wild birds have shown to be often infected by the same strains circulating in domestic flocks, representing a source of infection for poultry, especially in cases of poor confinement of reared animals, i.e., in backyard and free-range farms as well as in commercial farms where biosecurity measures are poorly or not properly implemented [10,61].

The current study suggests and underlines the role played by human activities in infectious diseases entry and spread on farms. This role may be mitigated by applying strict biosecurity measures along the whole production chain. The focus must be on changing and improving people’s behaviour in such a way that the risk of disease entry, spread, and transmission could be decreased consequently. The implementation of biosecurity measures, paying particular attention to vehicles and personnel movement in and out of farms and efficient and complete cleaning and disinfection operations, should go along with a strict surveillance on animal clinical status by recognizing signs and symptoms of infectious disease timely. Moreover, the use of suitable PPE remains essential not only to protect the health of farmers but also to reduce the risk of disease spread on farms. A great percentage of farmers/farm workers in the study declared not to use PPE in daily routine, and this represents a weak point that requires attention. In this sense, specific training activities to the staff of poultry farms organized by health authorities, veterinarians, professional associations, etc., would be strategic in order to give instructions and suggestions and to educate personnel on the importance of biosecurity measures aimed at reducing the risk of introduction and maintenance of these agents on farm environment [63].

Despite the limitations intrinsic to cross-sectional studies, we think that the external validity of the risk factor analysis of our study was not put at risk by the way the data were collected. Our dataset was not based on convenience sampling, as the recruited herds were obtained through stratified random sampling. Moreover, even if the presence of risk factors and outcomes were determined simultaneously by design, it is unlikely that the right temporal sequence (i.e., whether the exposure to the considered risk factors or the infection came first) can be misinterpreted.

However, the available sample size may have limited the achievable precision of the risk estimates, therefore preventing to statistically confirm the association with part of the candidate risk factors included in our bivariate analysis. New, larger studies in the future should further investigate their potential role.

## 5. Conclusions

*C. gallinacea* was confirmed to be the endemic chlamydial species in chickens in our territories, whereas *C. psittaci* was found only in backyard farms, in pigeons, and mixed-species farms.

The high prevalence of *Chlamydia spp.* in backyard flocks we detected paired with the fast-growing backyard chicken industries in many countries worldwide highlights the urgent need of specific legislation regarding strict biosecurity rules in this sector.

The significative *Chlamydia*-species correspondence proven in this study between animals and workers in the same farm, particularly strong for *C. gallinacea*, raises new questions that need to be addressed, especially related to the possible zoonotic potential of this species.

The results of the study yielded baseline information to address future epidemiologic, farm management, and public health policies for the prevention of chlamydial infection inside and outside Italy.

## Figures and Tables

**Table 1:** Number of farms and samples by holding size, reared species, and laboratory results.

Holding Size	Poultry Species	Sampled Farms	C*hlamydiaceae*-Positive Farms Ct (Min–Max) ^a^	*Chlamydia* Species Typing			Collected Samples	*Chlamydiaceae*-Positive Samples	*Chlamydia* Species-Typing PCR		
				*C. gallinacea*	*C. psittaci*	*C. avium*			*C. gallinacea*	*C. psittaci*	*C. avium*
Commercial farms ^b^	chicken	76	11 Ct (24–39.3)	10	-	-	1380	73	67	-	-
	duck	2	-	-	-	-	42	-	-	-	-
	turkey	1	-	-	-	--	16	-	-	-	-
	geese	1	-	-	-		16	-	-	-	-
	mixed poultry	3	-	-	-	-	64	-	-	-	-
Backyard farms ^c^	chicken	22	6 Ct (21.1–39.3)	6	-	-	348	37	33	-	-
	mixed species	7	5 Ct (27–38.9)	5	2	-	154	43	32	5	-
	pigeon	2	1 Ct (37.5–38.9)	-	1	-	43	2	-	2	-
Total		114	23	21 (10 ^b^ + 11 ^c^)	3 ^c^		2063	155	132	7	

^a^ rt-PCR Ct values from positive farms; ^b^ commercial farms > 250 animals raised; ^c^ backyard farms < 250 animals raised.

**Table 2:** Risk factors potentially associated with *Chlamydiaceae* presence in a farm. N, number of farms in the category; Pos, number of positive farms in the category; %, Pos/N; PR, prevalence ratio of exposed to non-exposed. Only the factors statistically significant are shown (i.e., 1 not included in the 95%CI).

Risk Factor	Exposure Level	N	Pos (%)	PR (95% CI)
Holding size	backyard	31	12 (38.7)	2.9 (1.3–6.6)
	commercial	83	11 (13.3)	1
Presence of free-range sheds	yes	29	13 (44.8)	4.1 (1.8–9.7)
	no	83	9 (11)	1
Presence of anti-sparrow nets	no	14	7 (50)	3.5 (1.4–8.6)
	yes	97	14 (14.4)	1
Full/empty cycles	no	26	10 (38.5)	3 (1.3–7.1)
	yes	86	11 (12.8)	1
Litter usage	no (only in free-range groups)	29	13 (44.8)	4.6 (1.9–11.1)
	yes	82	8 (9.8)	1
Feathers in the surroundings of the holding	yes (sometimes)	98	15 (15.3)	3 (1.1–8.2)
	no	11	5 (45.5)	1
Faeces in the surroundings of the holding	yes (sometimes)	5	3 (60)	3.8 (1.1–13.1)
	no	102	16 (15.7)	1
Bushes in the surroundings of the holding	yes (sometimes)	31	13 (41.9)	4.1 (1.7–10)
	no	79	8 (10.1)	1

**Table 3:** Matching between animal and human positivity to *Chlamydiaceae* within the same farm.

ID Human Sample	*Chlamydia* Species at Human Level	*Chlamydia* Species at Farm Level	Farms Holding Size	Poultry Species
22	*C. gallinacea*	*C. gallinacea*	Commercial	Chicken
36	*C. gallinacea*	*C. gallinacea*	Backyard	Chicken
37	*C. psittaci*	*C. psittaci*	Backyard	Pigeons
40	*Chlamydiaceae*	*C. gallinacea*	Commercial	Chicken
42	*C. gallinacea*	*C. gallinacea*	Commercial	Chicken
84	*C. gallinacea*	*C. gallinacea*	Commercial	Chicken
85	*C. gallinacea*	*C. gallinacea*	Commercial	Chicken
89	*C. gallinacea*	*C. gallinacea*	Commercial	Chicken
92	*C. gallinacea*	negative	Commercial	Chicken
98	*Chlamydiaceae*	*C. gallinacea*	Backyard	Chicken
99	*C. gallinacea*	*C. gallinacea*	Backyard	Chicken
176	*C. gallinacea*	negative	Commercial	Chicken
250	*Chlamydiaceae*	negative	Commercial	Chicken
252	*Chlamydiaceae*	*C. gallinacea*	Backyard	Mixed species
253	*C. gallinacea*	*C. gallinacea*	Backyard	Chicken
254	*C. gallinacea*	negative	Backyard	Mixed species

**Table 4:** Risk factors potentially associated with *Chlamydiaceae* positivity for occupational-exposed workers (bivariate PRs calculation), *n* = 113. N, number of workers in the category; Pos, number of positive workers in the category (%, Pos/N); PR, prevalence ratio of exposed to non-exposed. Table shows only the factors statistically significant (i.e., 1 not included in the 95%CI).

Risk Factor	Exposure Level	N	Pos (%)	PR (95%CI)
Type of farming	eggs production/reproduction	53	12 (22.6)	3.4 (1.1–10.5)
	broiler/meat production/mixed	60	4 (6.7)	1
Presence of *Chlamydiacea* on farm	yes	32	12 (37.5)	7.6 (2.4–23.5)
	no	81	4 (5)	1
Presence of free-range sheds	yes	30	9 (30)	3.5 (1.3–9.4)
	no	82	7 (8.5)	1
Full/empty cycles	no	24	8 (33.3)	3.6 (1.4–9.7)
	yes	87	8 (9.2)	1
Withdrawal of dead animals at the end of the cycle	no	36	10 (27.8)	3.5 (1.3–9.6)
	yes	75	6 (8)	1
Grass in the surroundings of the holdings	yes	9	4 (44.4)	3.8 (1.2–11.7)
	no	102	12 (11.8)	1
Various objects in the surroundings of the holdings holdings	yes	28	8 (28.6)	3 (1.1–7.9)
holdings	no	83	8 (9.6)	1
Bushes in the surroundings of the holdings	yes	37	11 (30)	5.4 (1.7–17)
	no	73	4 (5.5)	1
Year of sampling	2019	49	12 (24.5)	3.9 (1.3–12.1)
	2018	64	4 (6.3)	1

**Table 5:** MLST typing results on samples from farm 41645, 44638, 60260, and 67320 and two human samples. Samples in bold are presented in the phylogenetic tree in Figure 7.

FARM ID	Sample ID	MLST								
		*Chlamydiacea*	*gat*A	*opp*A	*hfl*X	*gid*A	*eno*A	*hem*N	*fum*C	ST
41645p ^1^	1	31.9								
41645p	2	31.6								
41645p	5	29.6								
41645p	8	28.4	44	36	38	45	36	30	28	313
41645p	9	30.5	44	36	38	45	36	30	28	313
44638p	9	29.6								
44638p	11	31.9	44	36	40	45	108	30	28	317
44638p	12	27.1								
44638p	13	32	44	36	40	45	108	30	28	317
44638p	16	32								
60260p	1	30.7								
60260p	2	30.8								
60260p	3	28	44	36	38	45	36	29	28	314
60260p	4	27.2	44	36	38	45	36	29	28	314
60260p	5	28.2								
60260p	6	30.9								
60260p	7	27	44	36	38	45	36	29	28	314
60260p	10	30.6								
67320p	1	25								
67320p	5	24.1	44	37	40	45	36	29	28	315
67320p	6	21.1								
67320p	7	22.6	44	37	40	45	36	29	28	315
67320p	9	30.2								
67320p	12	25.9								
67320p	13	27.8								
67320p	14	27								
67320p	15	28.2								
44638h ^2^	89		44	36	40	45	108	30	28	317
71895h	22		44	36	40	45	36	30	28	316

^1^ p, poultry samples; ^2^ h, human samples.

## Supplementary Materials

The following supporting information can be downloaded at: https://www.mdpi.com/article/10.3390/ijerph19042174/s1, Supplementary Material 1.
