# Supplementary material for: Chlamydia Species and Related Risk Factors in Poultry in North-Western Italy: Possible Bird-to-Human Transmission for C. gallinacea
Source: Int J Environ Res Public Health. 2022 Feb 15;19(4):2174. doi: 10.3390/ijerph19042174 (PMC8872282; doi:10.3390/ijerph19042174)
Supplement: Supplementary file 1 [file ijerph-19-02174-s001.zip › ijerph-1541526-supplementary/ijerph-1541526-supplementary S1/README_file.pdf]

International Journal of
Environmental Research and Public Health

Article

Chlamydia species and related risk factors in poultry in north-western Italy: possible bird-to-human transmission for C. gallinacea

SUPPLEMENTARY MATERIAL

README file

1) File “veterinary_database.dta”, n=114 farms

Variables reported in the dataset:

- “codaz”: unique farm code;
- “tipol”: farm holding size (“industriale”=commercial; “rurale/fam.”=backyard);
- “specie”: species reared;
- “orientam”: type of farming;
- “comune”: farm location;
- “tot_camp”: total number of samples collected in each farm;
- “n_pos”: total number of positive samples in each farm;
- “c_psittaci”: total number of positive samples positive for *C. psittaci* in each farm;
- “c_gallinacea”: total number of positive samples positive for *C. gallinacea* in each farm;
- “c_abortus”: total number of positive samples positive for *C. abortus* in each farm;
- “c_avium”: total number of positive samples positive for *C. avium* in each farm;
- “n_neg”: total number of negative samples in each farm;
- “az_pos”: positivity of the farm (0=no; 1=yes);
- “data”: date of sampling/administration of the questionnaire;
- “aa_costr”: year of construction of the farm;
- “aa_ristrut”: year of the last major renovation of the farm;
- “n_cap_murat”: number of masonry sheds;
- “n_tunnel”: number of tunnels;
- “cap_free_range”: are there some free-range sheds?
- “parch”: is there a parking area?
- “canc_sbarra”: is there a gate/bar for access control?

- “disinf_mezzi”: is there a disinfection platform/arch/pump for cleaning the vehicles at the entrance?
- “reti”: are there effective anti-bird nets in the openings of the sheds without external parquets?
- “segnal_mac”: have there been reports of the slaughterhouse in relation to well-being indicators towards the farm in the last 5 years?
- “cicli”: are full-full-empty cycles performed?
- “lettiera”: is the litter box used?
- “rit_morti”: does the withdrawal of dead animals take place at the end of the cycle?
- “vis_disinf”: during your last two visits to the farm did the disinfectant trays at the entrance to each shed contain the disinfectant?
- “vis_calz_cambio”: during your last two visits to the farm did the farm staff change footwear before entering each single shed?
- “vis_disp_indum”: during your last two visits to the farm did you notice if shoes and clothing dedicated to people authorized to access the facilities were available?
- “vis_indoss_indum”: during your last two visits to the farm did you notice if people authorized to access the facilities were asked to wear shoes and clothing dedicated to them?
- “circost_erba”: state of maintenance of the areas surrounding the holding: presence of grass;
- “circost_oggetti”: presence of various objects on the ground;
- “circost_piume”: presence of feathers;
- “circost_feci”: presence of faeces;
- “circost_alberi”: presence of trees;
- “circost_cesp”: presence of bushes;
- “vis_ratti”: during your last two visits to the farm did you notice direct/indirect evidence (e.g. faeces) of the presence of mice or other vermin?
- “vis_avifauna”: during your last two visits to the farm did you notice direct/indirect evidence (e.g. faeces) of wild bird life?
- “lett_stoc”: is the virgin litter stored in a covered and protected place?
- “lett_aggiunta”: is litter added during the cycle?
- “deratiz”: who manages the rodent control procedure?
- “pers_caccia”: does anyone among the staff who regularly work on the farm carry out hunting activities?
- “altri_cn_gt”: are there other pets? Dogs and cats;
- “altri_cv”: horses;
- “altri_bv”: cattle;
- “altre_cp_pc”: sheep and goats;
- “altri_sn”: pigs;
- “altri_vol”: other birds (in addition to those reared for commercial reasons);

- "km1_h2o": in the area near the farm (approximately 1 km) are there any water sources (rivers, ponds, wetlands, reserves)?
- "km1_boschi": in the area near the farm (approximately 1 km) are there any woods?
- "km1_allev": in the area near the farm (approximately 1 km) are there any other poultry farms (backyard or commercial)?
- "area_umida": is the farm located in a humid area, natural or artificial, permanent or temporary?

2) File "human_database.dta", n=145 farmers/farm workers/veterinary officers

Variables reported in the dataset:

- "codaz": unique farm code;
- "tipol": farm holding size ("industriale"=commercial; "rurale/fam."=backyard);
- "id": identification number of the sampled individual;
- "data_consenso": date of consent and sampling;
- "sintomi": clinical symptoms;
- "sintomi_inizio": onset of clinical symptoms;
- "viaggi_15gg": trips within the 15 days prior to the onset of clinical symptoms;
- "fuma": smoker yes/no;
- "allergie": allergies;
- "atb_15gg": antibiotic therapies in place in the last 15 days;
- "farmaci": drugs in use;
- "polmoniti": pneumonia;
- "ricoveri_1mese": hospitalizations in the last month;
- "condiz_med_pre": pre-existing medical conditions;
- "vacc_infl": seasonal flu vaccination;
- "attivita": type of activity carried out on the farm;
- "attiv_aa": years of activity;
- "contatto_spavic": direct contact with poultry species;
- "cont_tacch": contact with turkey;
- "cont_anatra": contact with duck;
- "cont_pollo": contact with chicken;
- "cont_oche": contact with geese;
- "cont_altro": contact with other poultry species;
- "cont_freq": contact frequency;
- "cont_15gg": direct contact in the last 15 days;

- “dpi_nessuno”: no use of personal protective equipment;
- “dpi_guanti”: use of protective gloves;
- “dpi_masch”: use of mask;
- “dpi_indum”: use of protective clothing;
- “abitaz_allev”: house near the farm;
- “clam_esito_h”: Chlamydia presence research outcome in human samples;
- “ct_clam_h”: quantitative outcome for Chlamydia presence research;
- “clam_spp_h”: outcome of the search for Chlamydia species in human samples;
- “ct_spp_h”: quantitative outcome for Chlamydia species research in human samples;
- “clam_spp_avicoli”: outcome of the search for Chlamydia species in poultry;
- “ct_avicoli”: quantitative outcome for Chlamydia species research in poultry.
